# Terroir Effect on the Phenolic Composition and Chromatic Characteristics of Mencía/Jaen Monovarietal Wines: Bierzo D.O. (Spain) and Dão D.O. (Portugal)

**DOI:** 10.3390/molecules25246008

**Published:** 2020-12-18

**Authors:** Fernanda Cosme, Alice Vilela, Luís Moreira, Carla Moura, José A. P. Enríquez, Luís Filipe-Ribeiro, Fernando M. Nunes

**Affiliations:** 1 CQ-VR, Chemistry Research Centre-Vila Real, Food and Wine Chemistry Lab, Biology and Environment Department, University of Trás-os-Montes and Alto Douro, 5001-801 Vila Real, Portugal; fcosme@utad.pt (F.C.); avimoura@utad.pt (A.V.); 2 CQ-VR, Chemistry Research Centre-Vila Real, Food and Wine Chemistry Lab, School of Life Sciences and Environment, University of Trás-os-Montes and Alto Douro, 5001-801 Vila Real, Portugal; al57742@utad.eu (L.M.); marina.prazelos@gmail.com (C.M.); apenri@gmail.com (J.A.P.E.); fmota@utad.pt (L.F.-R.); 3 CQ-VR, Chemistry Research Centre-Vila Real, Food and Wine Chemistry Lab, Chemistry Department, University of Trás-os-Montes and Alto Douro, 5001-801 Vila Real, Portugal

**Keywords:** red wine, Mencía, Jean, terroir, anthocyanins, phenolic acids, flavonols, wine color

## Abstract

‘Mencía’/‘Jaen’ it’s an important red grape variety, exclusive of the Iberian Peninsula, used in wine production namely in Bierzo D.O. and Dão D.O., respectively. This work evaluates the effect of the two different “terroirs” on the phenolic composition and chromatic characteristics of ‘Mencía’/‘Jaen’ monovarietal wines produced at an industrial scale in the same vintage. Using Principal Component Analysis (PCA), Partial Least Squares-Discrimination Analysis (PLS-DA), and Orthogonal PLS-DA (OPLS-DA) it was found that peonidin-3-coumaroylglucoside, petunidin-3-glucoside, malvidin-3-coumaroylglucoside, peonidin-3-glucoside, malvidin-3-acetylglucoside, malvidin-3-glucoside, and ferulic acid were the phenolic compounds with the highest differences between the two regions. PLS regression allowed to correlate the differences in lightness (L*) and redness (a*) of wines from ‘Jaen’ and ‘Mencía’ to differences in colored anthocyanins, polymeric pigments, total pigments, total anthocyanins, cyanidin-3-acetylglucoside, delphinidin-3-acetylglucoside, delphinidin-3-glucoside, peonidin-3-coumaroylglucoside, petunidin-3-glucoside and malvidin-3-glucoside in wines, and the colorless ferulic, caffeic, and coutaric acids, and ethyl caffeate. The wines a* values were more affected by colored anthocyanins, ferulic acid, total anthocyanins, delphinidin-3-acetylglucoside, delphinidin-3-glucoside and petunidin-3-acetylglucoside, and catechin. The positive influence of ferulic acid in the a* values and ferulic, caffeic, coutaric acids, and ethyl caffeate on the L* values can be due to the co-pigmentation phenomena. The higher dryness and lower temperatures during the September nights in this vintage might explain the differences observed in the anthocyanin content and chromatic characteristics of the wines.

## 1. Introduction

**Figure 1:**
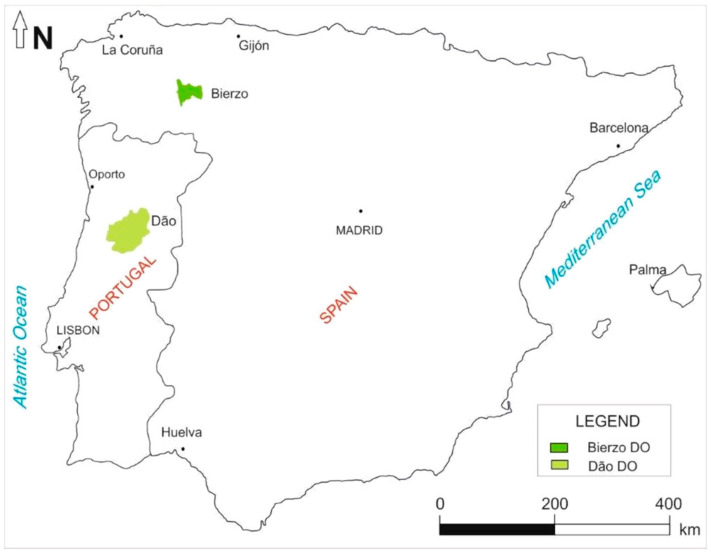
Location of Bierzo D.O. and Dão D.O. where ‘Mencía’ and ‘Jaen’ monovarietal wines are produced.

‘Mencía’ in Spain and ‘Jaen’ in Portugal is a grape variety present almost exclusively in the northwest of the Iberian Peninsula whose economic importance has been growing in the last years. ‘Mencía’ is extensively cultivated in Galicia, north-western Spain, to produce quality red wines with five Denomination of Origin (D.O.) Rías Baixas in the province of Pontevedra, Ribeiro, Valdeorras and Monterrei in the province of Ourense and Ribeira Sacra between the province of Ourense and Lugo. However, this grape variety also predominates in the Bierzo region (Figure 1) located in the northwest of the province of León (Castile and León, Spain) originating the ‘Mencía’ red wines with Bierzo D.O. [1,2]. In Portugal, this grape variety is known as ‘Jaen’ and is almost exclusively cultivated in the Dão Demarcated Region of Portugal (Figure 1) [1,2], whose importance for wine production in this region is growing, and a significant increase in the vineyard area of this grape variety between 2013 to 2017, from 1 731 ha [3] to 3 789 ha [3], respectively, has been observed.

The wine quality is determined by the conditions of the growing area, of the vintage, agricultural practices that will influence the grape composition, and by the winemaking technology used. The same variety, growing in two different viticultural regions with diverse climatic conditions, results in wines with different content of phenolic compounds in the same vintage [4]. Several factors such as soil type, environmental conditions, agricultural practices, climatic conditions, vine phenology, or winemaking processesall contribute to the “terroir” effect that can change the chemical composition of grapes and wines [5,6,7,8]. In the resolution OIV/VITI 333/2010 [9] the International Organization of Vine and Wine (OIV), defines vitivinicultural “terroir”-vitivinicultural “terroir” as a concept that refers to an area in which collective knowledge of the interactions between the identifiable physical and biological environment and applied vitivinicultural practices develop, providing distinctive characteristics for the products originating from this area. “Terroir” includes specific soil, topography, climate, landscape characteristics, and biodiversity features.

The phenolic, aroma, and mineral composition, as well as the sensory descriptors of red wines vinified with ‘Mencía’ grape variety, were studied by several authors. There are various works concerning the characterization of the aroma profile of ‘Mencía’ red wines [10,11,12,13,14,15,16], with an observed change in the ‘Mencía’ wine aroma profile depending on the grape’s geographic origin and vintage [17,18]. Also, pre-fermentative maceration techniques (enzymes, refrigeration, and cryomaceration) were shown to influence the aroma profile of ‘Mencía’ wines [19,20]. The use of indigenous yeasts has been used as a tool to increase the diversity of ‘Mencía’ monovarietal wines [21]. Other studies were performed on the content of minerals [22,23], and wine sensory descriptors [24].

Although phenolic compounds, including phenolic acids, anthocyanins, and tannins, contribute to wine color, bitterness, astringency, and antioxidant capacity [25,26], fewer studies were performed on the phenolic composition of ‘Mencía’ wines [27,28,29,30,31]. These studies did not include wines produced in the Bierzo D.O. and no studies were reported for the wines produced with Jaen grape variety in the Dão D.O.

It is expected that the geographical origin, related to the different edaphoclimatic conditions of the two regions, will impact differently on the composition of the wines and this information is of importance for the understanding of the enological potential and diversity of this grape variety in the two different regions. Therefore, the purpose of this work was to study the phenolic composition and chromatic characteristics of monovarietal ‘Mencía’ wines of the Bierzo D.O. and monovarietal ‘Jaen’ wines produced at the Dão D.O. at an industrial scale in the 2015 vintage to evaluate the impact of “terroir” on the wines produced from this important Iberian grape variety.

## 2. Results and Discussion

### 2.1. Effect of Bierzo D.O. and Dão D.O. “Terroirs” on the Anthocyanins and Phenolic Acid Composition of ‘Mencía’/‘Jaen’ Wines—Unsupervised Analysis

To study the effect of Bierzo D.O. and Dão D.O. “terroirs” on the phenolic composition of ‘Mencía’/‘Jaen’ monovarietal wines, the concentration of the individual phenolic compounds was determined by RP-HPLC-DAD (Reversed-Phase High-Performance Liquid Chromatography with Diode Array Detector, Table 1 and Table 2). In all wines analyzed gallic, *trans*-caftaric, coutaric isomer, coutaric, caffeic, *p*-coumaric, and ferulic acids along with the ethyl esters of caffeic and coumaric acids, and the flavanol catechin was present (Table 1). The concentrations determined for the phenolic acids, phenolic esters, and catechin agreed with those described by García-Falcón et al. [27] and Alén-Ruiz et al. [28] for ‘Mencía’ wines produced in the Galician Ribeiro D.O. (Ourense).

Eleven and thirteen anthocyanins were identified and quantified in the ‘Mencía’ red wine produced in the Bierzo D.O. and in the ‘Jaen’ red wine produced in the Dão D.O., respectively (Table 2). For the ‘Mencía’ wines, the anthocyanin composition and concentrations were in accordance with those obtained by Rivas-Gonzalo et al. [32], García-Falcón et al. [27], and Soto Vázquez et al. [29], although García-Falcón et al. [27] only detected seven anthocyanins at the end of malolactic fermentation.

**Figure 2:**
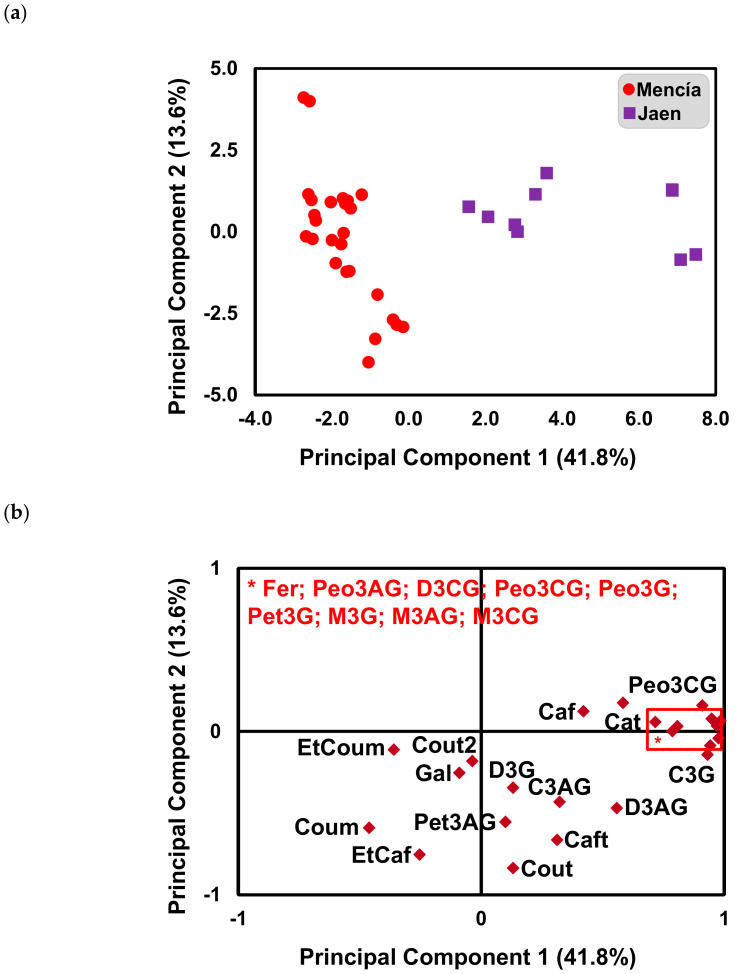
Sample scores projection on the first and second principal component (**a**) and variable loading on the first and second principal component (**b**) Delphinidin-3-glucoside (D3G), cyanidin-3-glucoside (C3G), peonidin-3-glucoside (Peo3G), petunidin-3-glucoside (Pet3G), malvidin-3-glucoside (M3G), delphinidin-3-acetylglucoside (D3AG), cyanidin-3-acetylglucoside (C3AG), peonidin-3-acetylglucoside (Peo3AG), petunidin-3-acetylglucoside (Pet3AG), malvidin-3-acetylglucoside (M3AG), delphinidin-3-coumaroylglucoside (D3CG), peonidin-3-coumaroylglucoside (Peo3CG), malvidin-3-coumaroylglucoside (M3CG), gallic acid (Gal), caftaric acid (Caft), coutaric acid (Cout), coutaric acid isomer (Cout2), caffeic acid (Caf), coumaric acid (Coum), catechin (Cat), ethyl ester of caffeic acid (EtCaf), ethyl ester of coumaric acid (EtCoum).

A principal component analysis (PCA) was applied to the anthocyanins and phenolic acids determined by HPLC-DAD (after normalization to zero mean and unit standard deviation) to reduce in an unbiased and unsupervised way the dimensionality of this multivariate dataset and identify the differences or similarities among the samples (Figure 2a) and to identify phenolic compounds responsible for the grouping or separation among the samples (Figure 2b). Figure 2a shows the score plot of the first two PC´s showing a clear separation of ‘Mencía’ and ‘Jaen’ wines. Additionally, replicate samples of the same label were grouped in the same cluster, although they were not overlapped, indicating that bottle to bottle variation occurred. The first PC (Principal Component), which explained 42% of the total variance of the original data set, correlated positively with malvidin-3-coumaroylglucoside (M3CG), petunidin-3-glucoside (Pet3G), malvidin-3-glucoside (M3G), malvidin-3-acetylglucoside (M3AG), peonidin-3-glucoside (Peo3G), cyanidin-3-glucoside (C3G), peonidin-3-coumaroylglycoside (Peo3CG), delphinidin-3-coumaroylglucoside (D3CG), and peonidin-3-acetylglucoside (Peo3AG). The second PC, which explained 14% of the total variance, correlated negatively with coutaric acid (Cout), ethyl ester of caffeic acid (EtCaf), caftaric acid (Caft), and coumaric acid (Coum) (Figure 2b). Therefore, the results from PCA allowed for the conclusion that ‘Jaen’ wine samples from Dão D.O. are separated from ‘Mencía’ wine samples from Bierzo D.O. according to PC1, showing a higher relative amount of M3CG, Pet3G, M3G, M3AG, Peo3G, C3G, Peo3CG, D3CG, and Peo3AG (Figure 2a,b).

**Figure 3:**
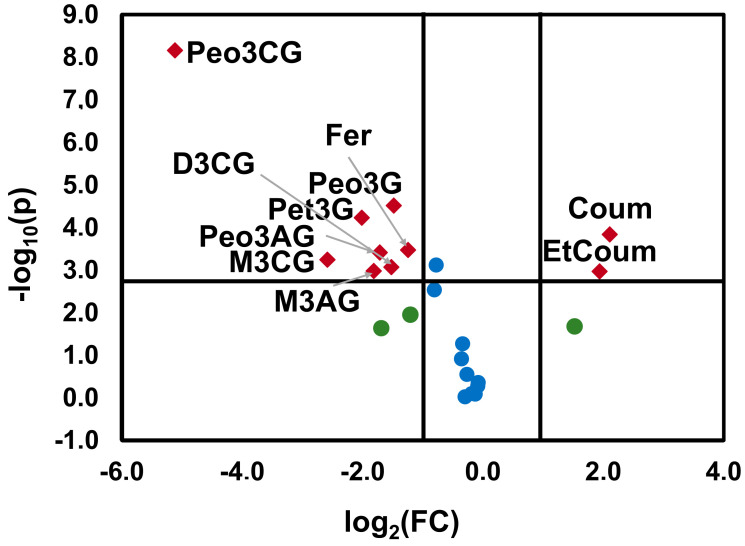
Volcano plot representing the statistical significance (*p*-values) on the Mann–Whitney non-parametric test and the fold change (FC) for the anthocyanins, phenolic acids, and catechin of wines ‘Mencía’/‘Jaen’ wines from Bierzo D.O. and Dão D.O., respectively. The horizontal line represents the threshold of significance corrected for multiple comparisons by Bonferroni’s method (*p* = 0.00217). Delphinidin-3-glucoside (D3G), cyanidin-3-glucoside (C3G), peonidin-3-glucoside (Peo3G), petunidin-3-glucoside (Pet3G), malvidin-3-glucoside (M3G), delphinidin-3-acetylglucoside (D3AG), cyanidin-3-acetylglucoside (C3AG), peonidin-3-acetylglucoside (Peo3AG), petunidin-3-acetylglucoside (Pet3AG), malvidin-3-acetylglucoside (M3AG), delphinidin-3-coumaroylglucoside (D3CG), peonidin-3-coumaroylglucoside (Peo3CG), malvidin-3-coumaroylglucoside (M3CG), gallic acid (Gal), caftaric acid (Caft), coutaric acid (Cout), coutaric acid isomer (Cout2), caffeic acid (Caf), coumaric acid (Coum), catechin (Cat), ethyl ester of caffeic acid (EtCaf), ethyl ester of coumaric acid (EtCoum).

To identify what phenolic compounds were more affected by the “terroir” of Bierzo D.O. and Dão D.O., a volcano plot was used (Figure 3), by plotting the negative logarithm of statistical significance (*p* values) of Mann–Whitney non-parametric test on the y-axis and the normal logarithm of the fold change on the x-axis. Bierzo D.O. wines contained significantly less M3CG, Pet3G, M3G, M3AG, Peo3G, C3G, Peo3CG, D3CG, and Peo3AG and significantly higher levels of Coum and EtCoum. These results supported the results obtained by PCA.

### 2.2. Effect of Bierzo D.O. and Dão D.O. Terroirs on the Anthocyanins and Phenolic Acid Composition of ‘Mencía’/‘Jaen’ Wines—Discrimination and Variable Importance

**Figure 4:**
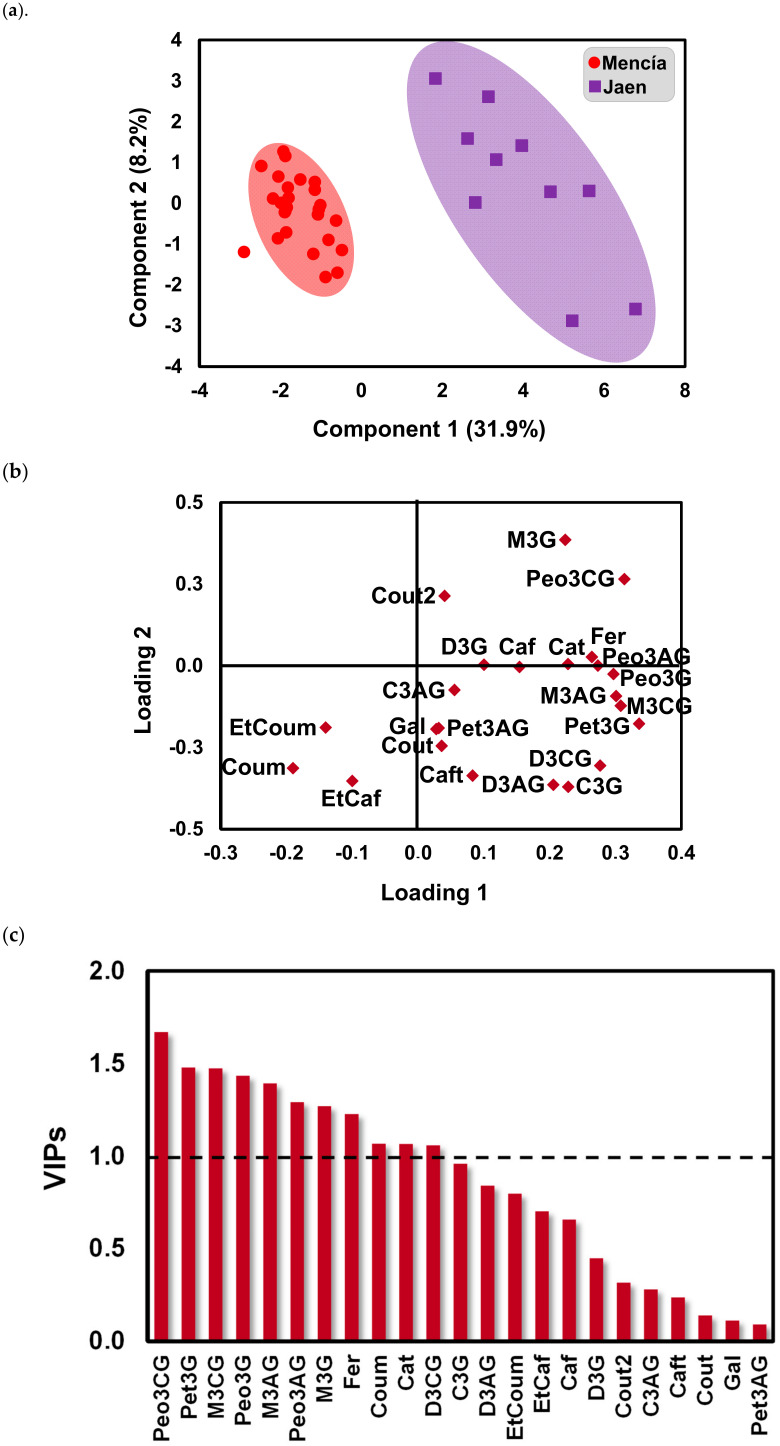
(**a**) Scores plot and (**b**) loadings plot of the first two factors of the Partial Least Squares-Discrimination Analysis (PLS-DA) model built with the phenolic compounds profile of the ‘Mencía’/‘Jaen’ monovarietal wines from the Bierzo D.O. and Dão D.O., respectively. Phenolic compounds ranked by variable in projection (VIP) scores (**c**). Delphinidin-3-glucoside (D3G), cyanidin-3-glucoside (C3G), peonidin-3-glucoside (Peo3G), petunidin-3-glucoside (Pet3G), malvidin-3-glucoside (M3G), delphinidin-3-acetylglucoside (D3AG), cyanidin-3-acetylglucoside (C3AG), peonidin-3-acetylglucoside (Peo3AG), petunidin-3-acetylglucoside (Pet3AG), malvidin-3-acetylglucoside (M3AG), delphinidin-3-coumaroylglucoside (D3CG), peonidin-3-coumaroylglucoside (Peo3CG), malvidin-3-coumaroylglucoside (M3CG), gallic acid (Gal), caftaric acid (Caft), coutaric acid (Cout), coutaric acid isomer (Cout2), caffeic acid (Caf), coumaric acid (Coum), catechin (Cat), ethyl ester of caffeic acid (EtCaf), ethyl ester of coumaric acid (EtCoum).

Partial least squares-discriminant analysis (PLS-DA) and orthogonal PLS-DA (OPLS-DA) were used to access their prediction efficiency and most importantly determine the main characteristics that distinguish the wines from the two terroirs concerning their phenolic composition. The PLS-DA model was developed using the standardized phenolic composition of ‘Mencía’ and ‘Jaen’ wines (Figure 4). Figure 4a shows the score plot of the first two components of the PLS-DA model obtained. It was similar to the PCA score plot (Figure 2a), however, the separation of wines according to geographical origin appears more obvious. This might be explained by the fact that the PLS-DA algorithm maximizes the variance between groups rather than within the group. The PLS-DA loadings for the calibration model (Figure 4b) were similar to those observed in the PCA analysis (Figure 2b). The accuracy, R^2^, and Q^2^ for the PLS-DA calibration model were 1, 0.9364, and 0.83821, respectively (two PLS latent variables). The calibration statistics indicated that the model developed was acceptable to classify new samples.

To access the variable importance in the discrimination of ‘Mencía’/‘Jaen’ wines, the variable in projection (VIP) was used (Figure 4c). According to the VIP results, Peo3CG, Pet3G, M3CGlc, Peo3G, M3AG, M3G, and Fer were the most influential variables in the discrimination between ‘Mencía’ and ‘Jaen’ wines from Bierzo D.O. and Dão D.O., respectively.

**Figure 5:**
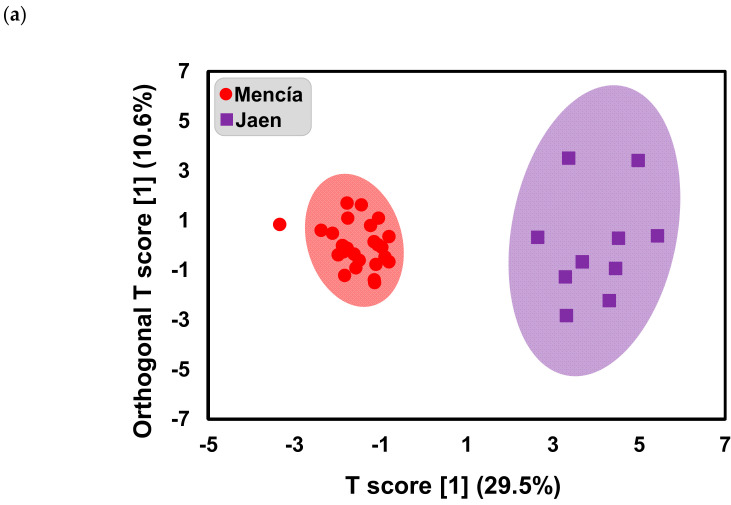

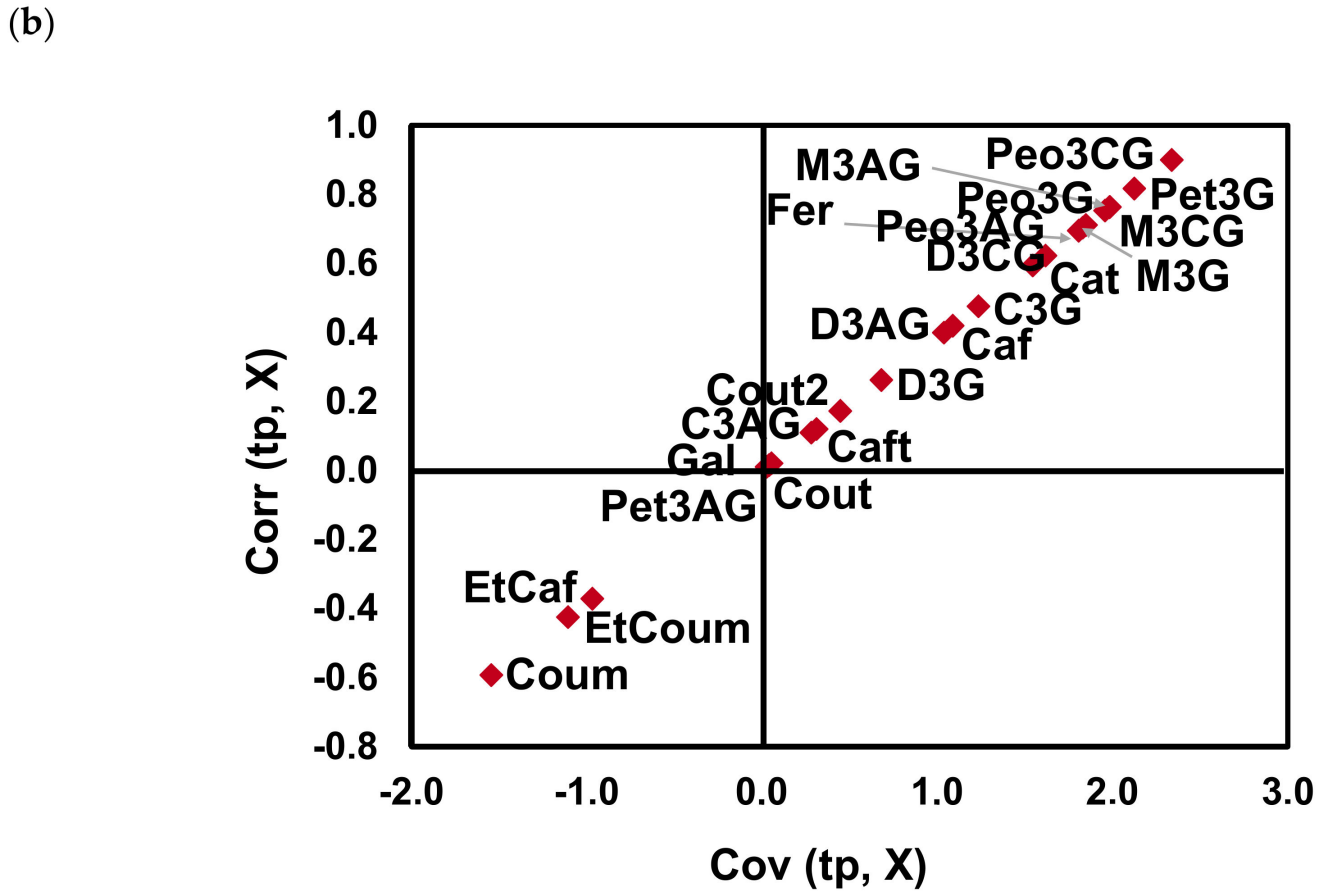
OPLS-DA (Orthogonal Partial Least Squares Discriminant Analysis) score plot for ‘Mencía’/‘Jaen’ monovarietal wines from Bierzo D.O. and Dão D.O. (**a**) and S-Plot for visualization of the variable influence on the OPLS-DA model by representing the covariance (Cov) and correlation (Corr) loading profiles resulting from the projection on the predictive component, tp (**b**). Delphinidin-3-glucoside (D3G), cyanidin-3-glucoside (C3G), peonidin-3-glucoside (Peo3G), petunidin-3-glucoside (Pet3G), malvidin-3-glucoside (M3G), delphinidin-3-acetylglucoside (D3AG), cyanidin-3-acetylglucoside (C3AG), peonidin-3-acetylglucoside (Peo3AG), petunidin-3-acetylglucoside (Pet3AG), malvidin-3-acetylglucoside (M3AG), delphinidin-3-coumaroylglucoside (D3CG), peonidin-3-coumaroylglucoside (Peo3CG), malvidin-3-coumaroylglucoside (M3CG), gallic acid (Gal), caftaric acid (Caft), coutaric acid (Cout), coutaric acid isomer (Cout2), caffeic acid (Caf), coumaric acid (Coum), catechin (Cat), ethyl ester of caffeic acid (EtCaf), ethyl ester of coumaric acid (EtCoum).

Generally, PCA and PLS-DA are used to distinguish samples and to explain the differences. When the number of variables were higher than the number of observations, PCA and PLS-DA models were suited to handle these data sets for discriminant analysis [33,34]. Nevertheless, for large data sets with a number of observations higher than the number of variables, PCA and PLS-DA scores and loadings can become rotated due to the presence in the data of strong systematic variations unrelated to the response, making more difficult the interpretation of the models. The OPLS-DA (Orthogonal partial least squares discriminant analysis) integrated an orthogonal signal correction filter to separate the variations in the data that are related to the prediction of a quantitative response from the variations not related or orthogonal to the prediction [35]. The advantage of OPLS-DA compared to PLS-DA is that the model is rotated so that class separation is found in the 1st predictive component, t_p_, also referred to as the correlated variation, and variation not related to class separation is seen in orthogonal components, t_o_, also referred to as the uncorrelated variation, facilitating model interpretation. The quality of the OPLS-DA model was validated by the values of the parameters R^2^X = 0.259, R^2^Y = 0.877, Q^2^ = 0.852, which demonstrated the potential usefulness of the OPLS-DA model (Figure 5).

Besides, in the OPLS-DA model, the S-plot was proposed as a tool that provides both the covariance and correlation loadings between the analytes and the t_p_, thus helping to identify both statistically and biochemically significant analytes [36]. In Figure 5b, the S-plot shows that peonidin-3-coumaroylglycoside (Peo3CG), petunidin-3-glucoside (Pet3G), peonidin-3-glucoside (Peo3G), malvidin-3-acetylglucoside (M3AG), and malvidin-3-coumaroylglucoside (M3CG) had relatively higher covariance and correlation loading values, and they were likely to be the key phenolic compounds for ´terroir’ differentiation.

**Figure 6:**
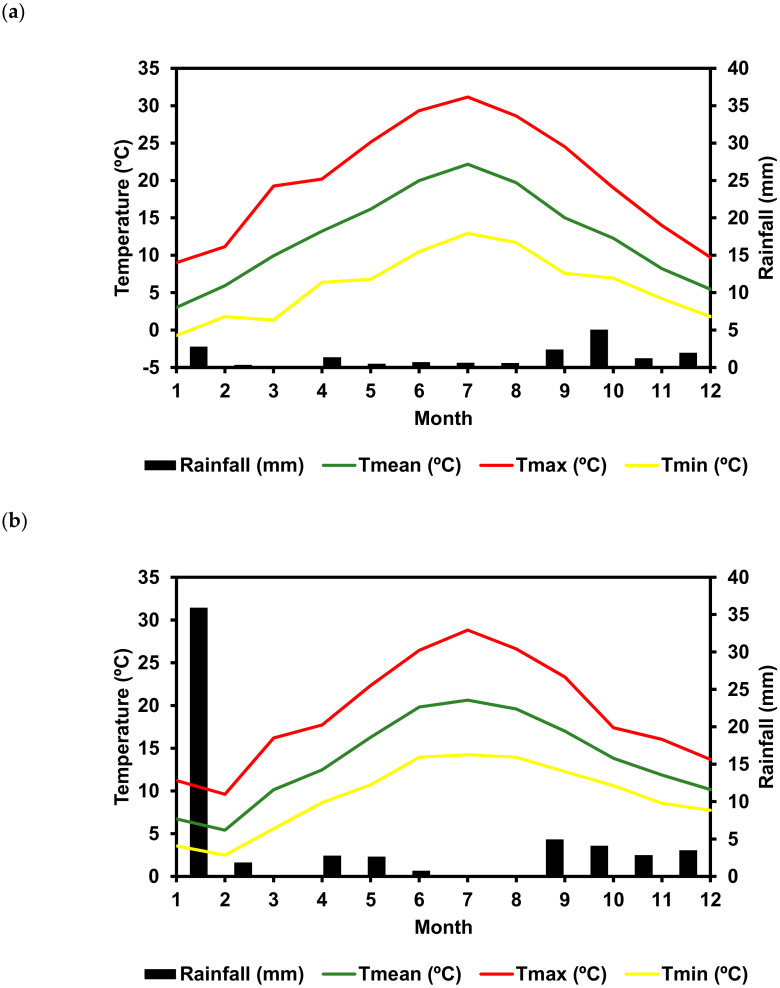
Distribution of rainfall and maximum (Tmax), minimum (Tmin), and mean temperatures (Tmean) in the (**a**) Bierzo D.O. and (**b**) Dão D.O. in 2015.

The composition of wines, especially the phenolic composition, is dependent on the chemical composition of grapes and on the winemaking technology used for its production. As wines analyzed in this work were from different wineries in both regions, and the differences observed between the two regions was significantly higher than the differences observed between the wines from each region, the winemaking technology used for wine production was not responsible for the differences observed in the phenolic composition of wines between the two regions. It has been shown that the main factors involved in terroir expression are soil, climate, and grape variety, and these factors interact [37]. Temperature and precipitation are the main factors influencing grapevine phenology, grape yield, as well as wine quality [38]. The wine composition produced in a particular region is influenced by the baseline climate, while the vintage effect is the result of climatic variability between vintages [39]. The monthly mean, minimum, and maximum temperature for this particular vintage (2015) in both regions are presented in Figure 6, along with the precipitation, and the bioclimatic indices commonly used for accessing the suitability of a particular region for wine production (Table 3). Winkler index (WI) measures the heat accumulation during the growing season [40], both regions are included in the Winkler’s Region II and classified as moderately cold. When considering the Huglin Heliothermic Index (HI) Dão D.O. corresponds to temperate warm and Bierzo to warm viticultural regions. The Dryness Index (DI) allows to access the availability of soil water content for the vine in the growing season, considering precipitation and reference evapotranspiration [41]. According to the values between April and October, Dao D.O. was a sub-humid region and Bierzo DO was a moderately dry region. The cool night index (CI) [42] considers the minimum temperatures during the grape maturation period, providing complementary information about the thermal regime in this period. The ripening stage is determining for the titratable acidity, pH, phenolic compounds, and anthocyanins as well for the flavor/aroma potential, and therefore CI is a reliable index for accessing the potential quality associated with viticultural climates [43]. Bierzo D.O. had CI values that correspond to very cool nights, whereas Dão D.O. had cool nights. The hydrothermic index of Branas, Bernon, and Levadoux (BI) [44] considers the influence of both temperature and precipitation on grape yield and wine quality. This index estimates the risk of downy mildew disease, which is a common limiting factor for grapevine yield [45]. Both regions presented a low risk of mildew disease. The bioclimatic indexes obtained for 2015 are in accordance with those described for Dão Region in the 1950–2000 period [46] and for the Bierzo region from 2007 up to 1967 [47], showing that 2015 was a typical climatic year. The major differences between the two regions in this particular vintage were the lower minimum temperature at night between April and September, being found a total number of days with a temperature below 10 °C during the maturation months (August and September) much higher in the Bierzo region (32 days) when compared to the Dão region (12 days). Also, the number of days with a maximum temperature above 30 °C was much higher in the Bierzo region (14 days) when compared to the Dão region (7 days). This different temperature profile between the two regions might explain the differences observed in the anthocyanins content of the wines from the two regions. In warmer climates, higher temperatures may result in negative changes in fruit composition. A significantly lower anthocyanins concentration at maturity was observed in grapevines exposed to 30 rather than 20 °C temperature treatments [48]. Another study found that high temperatures (35 °C) both inhibited anthocyanin production and degraded the anthocyanins that were produced [49]. High and low temperatures during ripening, likely affect the production and/or degradation of abscisic acid (ABA) in berry skins that affect the expression of VvmybA1 that controls the expression of the anthocyanin’s biosynthetic enzyme genes [50]. A day temperature of 35 °C completely inhibited anthocyanins synthesis in ‘Tokay’ berries, regardless of night temperature [51]. The effect of the weather variables in the phenolic composition of ‘Mencía’/‘Jaen’ wines were in accordance with the previous study of Vilanova et al. [18], who observed that the composition of ‘Mencía’ grapes, including the phenolic composition, was more affected by the vintage (from 2009 to 2012) than the geographic origin when studying the ‘Mencía’ cultivar located in different geographic areas from NW Spain (Amandi, Chantada, Quiroga-Bibei, Ribeiras do Sil, and Ribeiras do Miño).

### 2.3. Impact of the Phenolic Composition on the Color of Red Wines from ‘Mencía’/‘Jaen’

As expected from the phenolic composition of ‘Mencía’/‘Jaen’ wines, ‘Jaen’ wines from Dão D.O. presented a significantly higher color intensity when compared to ‘Mencía’ wines from Bierzo D.O. (14.1 vs. 11.3 a.u., *p* < 0.00687, respectively, Table 4). The color intensity for ‘Mencía’ wines from Bierzo D.O. obtained in this work was in accordance with the values described by Revilla et al. [52] for young red wines produced with ‘Mencía’ grapes in AOC Valdeorras (Galicia) (range from 6.43 to 17.21 a.u.), by García-Falcón et al. [27] also for ‘Mencía’ wines (range from 11.2 to 12.6 a.u.), by Bouzas-Cid et al. [16] (range from 9.1 to 10.8 a.u.), and by Soto Vázquez et al. [29] (range from 7.37 to 11.57 a.u.). However, Blanco et al. [21] obtained lower color intensity values for ‘Mencía’ monovarietal wines produced in Galicia (range from 6.87 to 7.67 a.u.).

As can be observed in Table 4, the ‘Jaen’ monovarietal wines from Dão D.O. presented a significantly lower lightness, L* value, when compared to the ‘Mencía’ wines from Bierzo D.O. (72.40 vs. 77.35, *p* < 0.02684, respectively). On the other hand, ‘Jaen’ monovarietal wines presented a significantly higher a* value (redness) than ‘Mencía’ wines (30.64 vs. 24.34, *p* < 0.00509, respectively). For the b* values (yellowness) there was no significant difference between ‘Jaen’ and ‘Mencía’ wines (7.70 vs. 6.53, *p* < 0.399, respectively).

**Figure 7:**
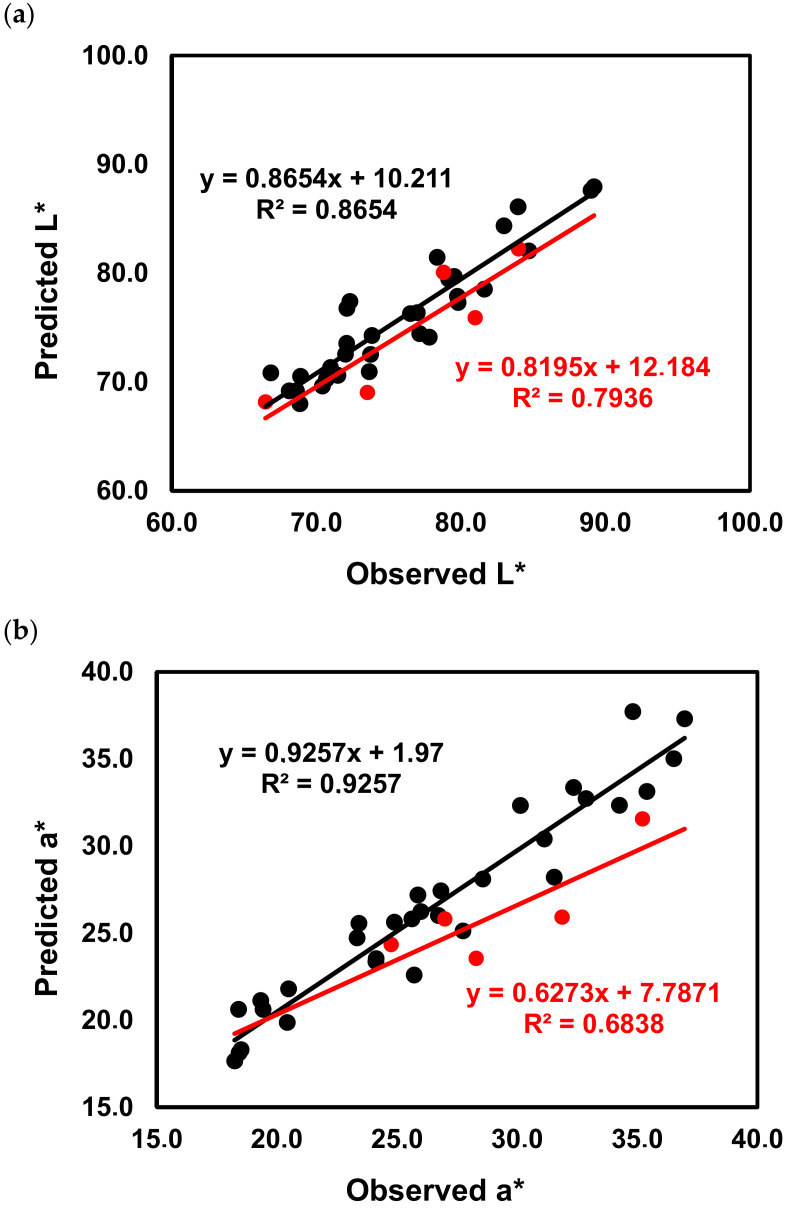
Calibration curves were obtained after Partial Least Squares (PLS) regression of the L* values (**a**) and of the a* values (**b**) of ‘Mencía’/‘Jaen’ wines using the phenolic composition as independent variables. Black circles calibration samples, red circles test samples.

To understand the relative contribution of the phenolic composition of ‘Mencía’ and ‘Jaen’ wines on the significantly different chromatic characteristics (L* and a* values), a Partial Least Squares (PLS1) regression of the standardized chromatic characteristics (Y) on the standardized phenolic and monomeric anthocyanins composition of wines (X) was performed. The number of factors for each dependent variable analyzed was estimated by 7-fold cross-validation and the prediction ability of the model obtained was determined using an independent validation set randomly selected. The regression curves obtained for L* and a* are presented in Figure 7a,b, respectively. For the L* value, 63.2% of the variance in the three PLS components related to phenolic and monomeric anthocyanin composition of wines explained 86.5% of the variation in L* values of ‘Mencía’/‘Jaen’ monovarietal wines with a Q^2^ cumulative of 0.6870. For the validation set, a R^2^ value of 0.7936 was obtained, showing a medium predictive ability. For the a* value, the phenolic composition and monomeric anthocyanin composition of ‘Mencía’/‘Jaen’ wines allowed to obtain a good model, with 69.6% of the variance in four PLS components related to the phenolic and monomeric anthocyanin composition of wines explaining 92.6% of the variation in a* values (Q^2^ cumulative = 0.7321). The predictive ability for the validation set was bad, showing that this model cannot accurately predict the a* value from the phenolic and monomeric anthocyanin composition of wines (Figure 7b).

This medium to the bad predictive ability of L* and a* values using only the individual phenolic composition of ‘Mencía’/‘Jaen’ red wines can be explained by the fact that in wine monomeric anthocyanins are involved in multiple equilibriums depending on the wine pH [53], co-pigmentation phenomenon [54], sulfur dioxide bleaching [55], and besides the monomeric anthocyanins, polymeric anthocyanins can also be present [56]. Therefore, although these are young wines and the monomeric anthocyanins are the most abundant anthocyanins, probably the wine’s chromatic characteristics cannot be described solely by the levels of the individual monomeric anthocyanins present. Therefore, the classic colorimetric methods of polymeric pigments, colored anthocyanins, total pigments, and total anthocyanins were added to the X matrix (Table 5) to access if these variables could increase the explanation of the dependent variables, the wines L* and a* values. 

**Figure 8:**
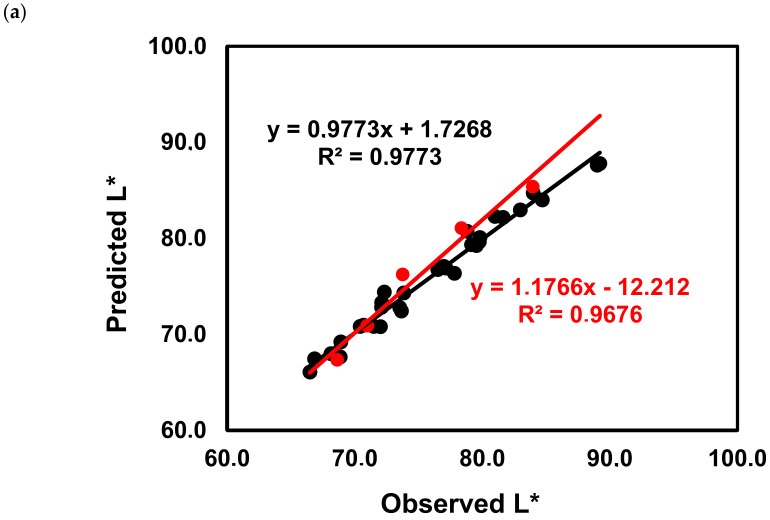

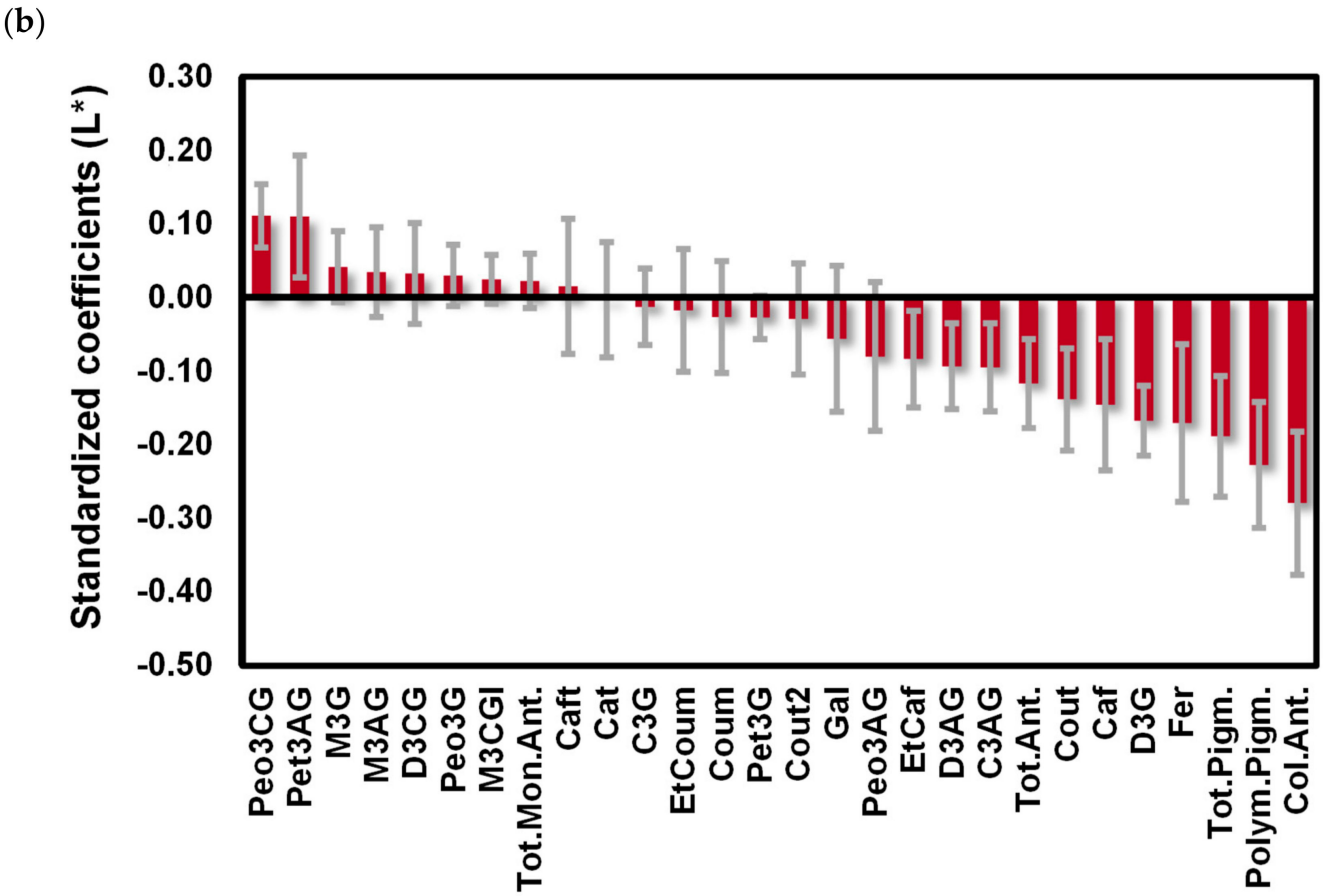
Calibration curves obtained after PLS regression of the L* values of ‘Mencía’/‘Jaen’ wines using the phenolic composition and colorimetric determination of colored anthocyanins, polymeric pigments, total pigments, total anthocyanins as independent variables (black circles calibration samples, red circles test samples) (**a**) and B coefficients plots (**b**). Delphinidin-3-glucoside (D3G), cyanidin-3-glucoside (C3G), peonidin-3-glucoside (Peo3G), petunidin-3-glucoside (Pet3G), malvidin-3-glucoside (M3G), delphinidin-3-acetylglucoside (D3AG), cyanidin-3-acetylglucoside (C3AG), peonidin-3-acetylglucoside (Peo3AG), petunidin-3-acetylglucoside (Pet3AG), malvidin-3-acetylglucoside (M3AG), delphinidin-3-coumaroylglucoside (D3CG), peonidin-3-coumaroylglucoside (Peo3CG), malvidin-3-coumaroylglucoside (M3CG), gallic acid (Gal), caftaric acid (Caft), coutaric acid (Cout), coutaric acid isomer (Cout2), caffeic acid (Caf), coumaric acid (Coum), catechin (Cat), ethyl ester of caffeic acid (EtCaf), ethyl ester of coumaric acid (EtCoum), total monomeric anthocyanins (Tot.Mon.Ant.), total pigment (Tot.Pigm.), polymeric pigments (Polym.Pigm.), colored anthocyanins (Col.Ant.), total anthocyanins measured colorimetrically (Tot.Ant.).

As can be observed in Figure 8a, the model quality for predicting the wine L* values and especially the wine a* values increased both in the calibration, and more importantly on the validation test sets. From the analysis of the standardized coefficients (Figure 8b), it can be observed that the variables with the highest influence in predicting the wines L* values were colored anthocyanins, polymeric pigments, total pigments, total anthocyanins C3AG, D3AG, D3G, PeoCG, Pet3G and M3G, but also the colorless Fer, Caf, Cout, EtCaf.

**Figure 9:**
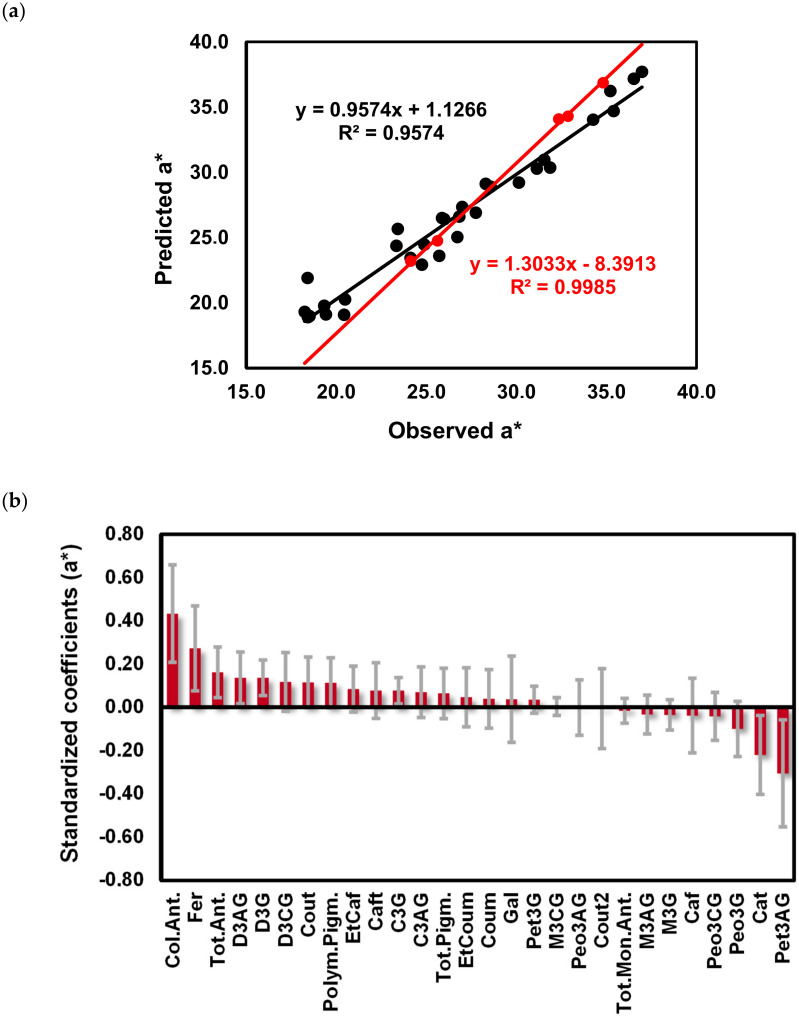
Calibration curves obtained after PLS regression of the a* values of ‘Mencía’/‘Jaen’ wines using the phenolic composition and colorimetric determination of colored anthocyanins (Col.Ant.), polymeric pigments (Polym.Pigm.), total pigments (Tot.Pigm.), total anthocyanins (Tot.Ant.) as independent variables (black circles calibration samples, red circles test samples) (**a**) and B coefficients plots (**b**). Delphinidin-3-glucoside (D3G), cyanidin-3-glucoside (C3G), peonidin-3-glucoside (Peo3G), petunidin-3-glucoside (Pet3G), malvidin-3-glucoside (M3G), delphinidin-3-acetylglucoside (D3AG), cyanidin-3-acetylglucoside (C3AG), peonidin-3-acetylglucoside (Peo3AG), petunidin-3-acetylglucoside (Pet3AG), malvidin-3-acetylglucoside (M3AG), delphinidin-3-coumaroylglucoside (D3CG), peonidin-3-coumaroylglucoside (Peo3CG), malvidin-3-coumaroylglucoside (M3CG), gallic acid (Gal), caftaric acid (Caft), coutaric acid (Cout), coutaric acid isomer (Cout2), caffeic acid (Caf), coumaric acid (Coum), catechin (Cat), ethyl ester of caffeic acid (EtCaf), ethyl ester of coumaric acid (EtCoum), total monomeric anthocyanins (Tot.Mon.Ant.), total pigment (Tot.Pigm.), polymeric pigments (Polym.Pigm.), colored anthocyanins (Col.Ant.), total anthocyanins measured colorimetrically (Tot.Ant.).

For the a* values of wines, also the most important variables in its prediction were the colored anthocyanins, Fer, total anthocyanins, D3AG, D3G and Pet3AG, Cat (Figure 9b). 

The positive influence of Fer in the a* values and Fer, Caf, Cout, EtCaf on the L* values can be due to the co-pigmentation phenomena of anthocyanins. Recent research assigned more relevance concerning co-pigmentation to hydroxycinnamic acid derivatives than flavan-3-ols. [57,58]. The latter result suggests that the levels of co-pigments in red wine are at least as important as the levels of anthocyanins in determining the differences in the color of red wines from ‘Mencía’/‘Jaen’ from Bierzo D.O and Dão D.O. in the 2015 vintage.

## 3. Materials and Methods

### 3.1. Wine Samples

Thirteen ‘Mencía’ red wine samples were from the Bierzo D.O located in the northwest of the province of León (Castile and León, Spain), and five ‘Jaen’ wines samples from the Dão D.O. All of them produced in Vintage 2015 in different wineries at an industrial scale and analyzed in 2016. The number of wine samples from each region was the one that the producers certified that the wines were exclusively produced from ‘Mencía’/‘Jaen’ grape varieties. Each producer supplied two bottles of wine.

### 3.2. High-Performance Liquid Chromatography (HPLC) Analysis of Anthocyanins, Catechin, and Phenolic Acids

The phenolic profile was performed by HPLC-DAD using an Ultimate 3000 HPLC system (Dionex Corporation, Sunnyvale, CA, USA) equipped with a photodiode array detector (PDA-100, Dionex Corporation, Sunnyvale, CA, USA). An ACE 5 C-18 column (250 × 4.6 mm) was used. The eluent was constituted by 5% aqueous formic acid (solvent A) and methanol (solvent B). The elution program was as follows: 5% of B from zero to 5 min followed by a linear gradient up to 65% of B until 65 min and from 65 to 67 min down to 5% of B. The photodiode detector assembly was operated between 200–600 nm and the chromatographic profile was recorded at 280, 325, and 525 nm. Then, 50 µL of the sample was injected at a flow rate of 1 mL/min and then the column was maintained at a temperature of 35 °C [59]. Quantification was performed with calibration curves with standard caffeic acid, coumaric acid, ferulic acid, gallic acid, and catechin. *Trans*-caftaric acid, 2-*S*-glutathionylcaftaric acid (GRP), and caffeic acid ethyl ester were expressed as caffeic acid equivalents, coutaric acid and coumaric acid ethyl ester were expressed as coumaric acid equivalents. A calibration curve of malvidin-3-glucoside, peonidin-3-glucoside, and cyanidin-3-glucoside was used for quantification of these anthocyanins. Using the coefficient of molar absorptivity (ε) and by extrapolation, it was possible to obtain the slopes for delphinidin-3-glucoside, petunidin-3-glucoside, and malvidin-3-coumaroylglucoside and perform the quantification. The results of delphinidin-3-acetylglucoside, petunidin-3-acetylglucoside, peonidin-3-acetylglucoside, cyanidin-3-acetylglucoside, and cyanidin-3-coumaroylglucoside were expressed as respective glucoside equivalents [60,61]. Analysis of each bottle were performed in duplicate.

### 3.3. Color Intensity, Total Anthocyanins, Colored Anthocyanins, Pigments, and Chromatic Characteristics

Red wine color intensity (A_420 nm_ + A_520 nm_ + A_620 nm_) and hue (A_420 nm_/A_520 nm_) were quantified as described in the OIV methods [62]. The concentration of total anthocyanins from red wine was determined by the SO_2_ bleaching procedure using the method described by Ribéreau-Gayon and Stronestreet [63], and the colored anthocyanin’s (A_520_ × 10 − A_520_^SO2^ × 10), total pigments (A_520_^HCl^ × 101) and polymeric pigments (A_520_ bis × 10) from red wine were determined according to Somers and Evans [64]. For the chromatic characteristics of red wine, the absorption spectra of wine samples were scanned from 380 to 780 nm using a 1-cm path length quartz cell, and the wines’ chromatic characteristics L∗ (lightness), a∗ (redness), and b∗ (yellowness) coordinates were calculated using the CIELab method according to OIV [62]. The Chroma (C* = [(a*)^2^ + (b*)^2^]^1/2^]) and hue-angle (^o^h = tang^−1^(b*/a*)) values were also determined. Analysis of each bottle were performed in duplicate.

### 3.4. Climacteric Data, Bioclimatic Indexes, and Soil Characteristics

The climacteric data of Bierzo (Spain) and Dão (Portugal) for the 2015 harvesting year were obtained from Ponferrada-Carracedelo (Spain, latitude 42°36′13″, longitude 6°30′02″, elevation above sea level of 800 m) and Viseu (Portugal, latitude 40°39′39″, longitude 7°54′34″, elevation above sea level 469 m), respectively, and included the maximum (Tmax), minimum (Tmin), and mean (Tmean) daily temperature along with the daily precipitation (P) [65,66]. To simplify the global description of weather conditions in both locations during the growing season in the two regions, the Winkler Index (WI) [40], Huglin Heliothermal Index (HI) [67], Cool Night Index (CI) [42], Dryness Index [41], and Branas hydrothermic index [44] were calculated, which quantify the impact of weather by single aggregate values.

The Winkler Index (WI) is based on heat summation or growing degree-days exceeding the threshold of 10 °C during the growing season (April 1 through October 31), calculated according to the following Equation (1):

(1) $$WI=\overset{30/10}{\underset{1/04}{\sum }}\frac{\left(T_{max}-T_{min}\right)}{2}-10$$

Negative values are calculated as zero when the addition is performed. According to the WI, geographical areas are divided into five climate regions: Region I (cold) WI ≤ 1390; Region II (moderately cold) 1391 ≤ WI ≤ 1670; Region III (warm) 1671 ≤ WI ≤ 1940: Region IV (moderately warm) 1941 ≤ WI ≤ 2220; Region V (hot) WI > 2200.

The Huglin Heliothermal Index (HI) is a heat summation index that takes daily mean temperature and daily maximum temperature into account as well as an adjustment for day length. It is calculated by summing the values from April 1 to October 31 using the following Equation (2):

(2) $$HI=\overset{31/10}{\underset{1/04}{\sum }}\frac{\left(T_{mean}-10\right)+\left(T_{max}-10\right)}{2}d$$

where *d* is the length of day coefficient ranging from 1.02 to 1.06 between 40° and 50° of latitude. Viticultural zones can be classified as [42]: Very cool (HI − 3; HI ≤ 1500); Cool (HI − 2; 1500 <HI ≤ 1800); Temperate (HI − 1; 1800 < HI ≤ 2100); Temperate warm (HI + 1; 2100 < HI ≤ 2400); Warm (HI + 2; 2400 <HI ≤ 3000); Very warm (HI + 3; HI > 3000).

Dryness Index (DI) indicates the potential water availability in the soil, related to the level of dryness in a region. DI is calculated according to the following Equation (3):

(3) $$W=\overset{31/10}{\underset{1/04}{\sum }}\left(W_{0}+P-Tv-Es\right)$$

where *W*_0_ is soil water reserve, *P* is precipitation, *Tv* is potential transpiration, and *Es* is soil evaporation (all variables in mm). Daily variables are used for this calculation, being distinguished four climate classes: very dry, where viticulture is limited by severe dryness (DI + 2: DI ≤ −100 mm); moderately dry (−100 < DI ≤ 50 mm); sub-humid (50 < DI ≤ 150 mm); and humid (DI > 150 mm) (defined in Tonietto and Carbonneau [42]). *W* is the estimate of soil water reserve at the end of the 1 April–31 October at modelled growing season period. To compute *Tv* and *Es* it is also necessary to compute the monthly total potential evapotranspiration. This was approximated by the Hargreaves method, which produces comparable results in arid and semiarid environments and requires temperature data only [68]. The result is mm of water in the soil. The initial *W*_0_ is usually taken as 200 mm [42,69].

The Cool Night Index (CI) is a night coolness variable determined using Equation (4):

(4) $$\mathrm{CI}=\overset{30/9}{\underset{1/09}{\sum }}\frac{T_{min}}{30}$$

According to the CI values, viticultural zones can be classified as [42]: very cool nights (CI + 2; CI ≤ 12); cool nights (CI + 1: 12 < CI ≤ 14); temperate nights (CI − 1: 14 < CI ≤ 18); warm nights (CI − 2: CI > 18).

The hydrothermic index of Branas, Bernon, and Levadoux (*BI*) [44] is the sum of the products of monthly mean temperature and monthly accumulated precipitation amount (in mm) during the April to August season (Equation 5):

(5) $$BI=\overset{30/08}{\underset{1/04}{\sum }}T_{mean}P$$

This index estimates the risk of downy mildew disease, which is a common limiting factor for grapevine yield [43]. This risk is usually considered low when BI values are below 2500 °C mm, high for values higher than 5100 °C mm, and very high for values higher than 7500 °C mm [70]. 

In Bierzo DO region, the predominant composition of the vine soils is a mixture of quartzites, sandstones, limestones, clays, and shales. The texture of the soil is predominantly clay-loam. The soil acidity ranged from 4–8.5 with levels above 6 in the valleys. The calcium oxide content is low and with organic material reaching 1% [71]. In the DO Dão, 97.4% of the soils are granitic, and the vineyards to produce DO Dão wines must be installed predominantly on granitic soils with brown non-humic litholic soils and in schist outcrops with brown non-humic Mediterranean soils. The soils presented an acid pH, poor in organic material and extractable mineral, with poor water retention capacity and, therefore, with low fertility [72,73].

### 3.5. Statistical Analysis

For statistical analysis of the chemical data, a one-way analysis of variance (α = 0.05) was applied. When this test was significant, means were compared using the Tukey test, using the STATISTICA 2010 software 10 (StatSoft, Tulsa, OK, USA) program.

PCA is a chemometric method for data reduction and exploratory analysis of high-dimensional data sets. PCA decomposes the original matrix into the multiplication of loading (the phenolic composition of wines) and score (wine samples) matrices. The principal components are linear combinations of the original variables. The principal components are uncorrelated and account for the total variance of the original variables. PCA is an unsupervised method of pattern recognition in the sense that no grouping of the data must be known before the analysis. The new sub-space defined by the principal components leads to a model that is easier to interpret than the original data set. From these results, it should be possible to highlight several characteristics and correlate them to the chemical composition of the different wine samples analyzed. Partial least squares-discriminant analysis (PLS-DA) is a regression method commonly used in multivariate statistics, to establish the relationship between 2 data information sets, referred to as X being the phenolic compounds of the wines, and Y, a binary vector value. Orthogonal PLS-DA (OPLS-DA) is a modification of PLS-DA, which separates the systematic variation in X into 2 parts, one is linearly related to Y (t_p_) and the other is orthogonal to Y (to). The quality of the OPLS-DA model is evaluated by the goodness-of-fit parameter (R^2^X), the proportion of the variance of the response variable that is explained by the model (R^2^Y), and the predictive ability parameter (Q^2^), which is calculated by a 7-round internal cross-validation of the data, using a default option of MetaboAnalyst 3.0. The parameters R^2^X and R^2^Y represent the fraction of the variance of matrix X and matrix Y, respectively, and Q^2^ represents the predictive accuracy of the model. R^2^X, R^2^Y, and Q^2^ values close to 1 indicate an excellent OPLS-DA model, and values higher than 0.5 indicate an OPLS-DA model of good quality [74]. In addition to the evaluation of the OPLS-DA models by calculating their R^2^X, R^2^Y, and Q^2^ values, we also analyzed more wine samples for further model validation. These samples were different from the wine samples used for model establishment. By calculating the recognition degree between wine samples used for model establishment and validation, we could validate the practical authenticating ability of these OPLS-DA models. The S-plot reflects the variable influence in an OPLS-DA model, which combines the covariance (magnitude) and correlation (reliability) loading profiles correlated with the predictive component in X (t_p_). In our research, analytes (phenolic compounds) that have higher covariance and correlation values also have higher concentrations and more repetitions in different samples during the OPLS-DA modelling, respectively. Using S-plot analysis, we can find out which analytes play a role both statistically significant (with high covariance values) and potentially biochemically significant (with high correlation values) in differentiating wine samples [36]. The selection of key phenolic compounds in sample differentiation needs to consider both covariance and correlation values.

## 4. Conclusions

Although the results obtained in this study correspond only to the 2015 vintage, they clearly show that the ‘Mencía’ monovarietal wines from Bierzo D.O. and the ’Jean’ monovarietal wines from Dão D.O. produced at an industrial scale presented a significantly different phenolic composition. The most significant differences between the wines from the two regions were related to the levels of peonidin-3-coumaroylglucoside, petunidin-3-glucoside, malvidin-3-coumaroylglucoside, peonidin-3-glucoside, malvidin-3-acetylglucoside, malvidin-3-glucoside, and ferulic acid. These differences resulted in wines with significantly different chromatic characteristics, with ‘Mencía’ wines presenting a significantly higher lightness and lower red color. For the L* value, the differences observed were mainly related to the colored anthocyanins, polymeric pigments, total pigments, total anthocyanins, cyanidin-3-acetylglucoside, delphinidin-3-acetylglucoside, delphinidin-3-glucoside, peonidin-3-coumaroylglucoside, petunidin-3-glucoside, and malvidin-3-glucoside, but also to the colorless ferulic acid, caffeic acid, coutaric acid, and ethyl ester of caffeic acid. For the wines, a* values, also the most important variables were the colored anthocyanins, ferulic acid, total anthocyanins, delphinidin-3-acetylglucoside, delphinidin-3-glucoside, and petunidin-3-acetylglucoside and catechin. The positive influence of ferulic acid in the a* values and ferulic acid, caffeic acid, coutaric acid, and ethyl ester of caffeic acid in the L* values can be due to the co-pigmentation phenomena of anthocyanins. These differences in the phenolic composition, namely in the anthocyanin content wines produced with ‘Mencía’/‘Jean’ grape variety in Bierzo D.O. and Dão D.O., respectively, can be explained by the different climacteric conditions of these two “terroirs” in 2015, with Bierzo D.O. presenting the characteristics of a warm, moderately dry viticultural region with very cold nights and Dão D.O. presenting the character of a temperate warm, sub-humid viticultural region with cold nights. 

## Figures and Tables

**Table 1:** ‘Mencía’ and ‘Jaen’ red wines phenolic profile (phenolic acid and catechin) expressed in mg/L.

Wine Samples	Gallic Acid	Catechins	*trans*-Caftaric Acid	Coutaric Acid Isomer	Coutaric Acid	Caffeic Acid	*p*-Coumaric Acid	Ferulic Acid	Caffeic Acid Ethyl Ester	Coumaric Acid Ethyl Ester
Mencía	21.42 ± 0.02 ^d^	8.14 ± 0.27 ^ab^	33.77 ± 0.43 ^d^	0.83 ± 0.01 ^b^	21.00 ± 0.21 ^g^	2.95 ± 0.94 ^ab^	5.63 ± 0.16 ^g^	0.81 ± 0.03 ^ab^	2.22 ± 0.10 ^e^	0.41 ± 0.00 ^a^
Mencía	20.76 ± 0.01 ^d^	7.28 ± 0.47 ^a^	5.10 ± 0.02 ^a^	1.25 ± 0.01 ^c^	5.63 ± 0.00 ^b^	3.96 ± 0.01 ^b^	2.58 ± 0.02 ^d^	2.40 ± 0.00 ^d^	1.40 ± 0.14 ^d^	2.06 ± 0.06 ^c^
Mencía	7.93 ± 0.10 ^ab^	7.10 ± 0.07 ^a^	35.42 ± 0.58 ^d^	0.60 ± 0.03 ^a^	19.76 ± 0.35 ^fg^	2.19 ± 0.30 ^a^	3.38 ± 0.04 ^e^	3.22 ± 0.02 ^e^	2.93 ± 0.07 ^f^	0.51 ± 0.33 ^ab^
Mencía	19.54 ± 0.34 ^cd^	8.79 ± 2.38 ^b^	17.28 ± 3.29 ^b^	0.56 ± 0.07 ^a^	9.83 ± 0.29 ^c^	2.38 ± 0.05 ^a^	5.78 ± 0.10 ^f^	0.92 ± 0.01 ^ab^	1.27 ± 0.02 ^d^	2.72 ± 0.09 ^d^
Mencía	17.46 ± 0.44 ^c^	2.37 ± 0.00 ^a^	34.37 ± 0.39 ^c^	0.72 ± 0.00 ^b^	13.29 ± 0.19 ^d^	3.78 ± 0.05 ^b^	0.86 ± 0.01 ^b^	0.66 ± 0.01 ^a^	0.40 ± 0.01 ^ab^	0.51 ± 0.02 ^a^
Mencía	21.88 ± 0.33 ^d^	6.11 ± 0.18 ^a^	36.50 ± 2.39 ^e^	1.30 ± 0.13 ^c^	19.91 ± 1.26 ^fg^	2.91 ± 0.018 ^ab^	4.01 ± 0.26 ^f^	1.62 ± 0.13 ^c^	1.43 ± 0.19 ^d^	2.38 ± 0.14 ^cd^
Mencía	21.61 ± 0.03 ^d^	7.26 ± 80.71 ^a^	29.78 ± 1.16 ^d^	0.73 ± 0.01 ^b^	15.77 ± 0.63 ^e^	2.80 ± 0.15 ^a^	1.31 ± 0.04 ^c^	2.70 ± 0.10 ^d^	1.02 ± 0.15 ^c^	0.65 ± 0.03 ^b^
Mencía	6.16 ± 0.35 ^a^	11.40 ± 0.85 ^bc^	4.90 ± 0.24 ^a^	0.33 ± 0.04 ^a^	1.68 ± 0.42 ^a^	2.13 ± 0.52 ^a^	1.59 ± 0.01 ^c^	0.79 ± 0.00 ^ab^	0.11 ± 0.07 ^a^	0.47 ± 0.02 ^a^
Mencía	22.55 ± 2.25 ^d^	10.35 ± 1.27 ^b^	30.24 ± 0.12 ^d^	0.81 ± 0.00 ^b^	16.34 ± 0.08 ^e^	2.82 ± 0.01 ^a^	4.28 ± 0.14 ^f^	0.61 ± 0.05 ^a^	0.94 ± 0.09 ^c^	0.58 ± 0.04 ^b^
Mencía	29.04 ± 2.75 ^e^	10.28 ± 3.52 ^b^	18.14 ± 0.38 ^b^	0.69 ± 0.17 ^ab^	6.91 ± 0.23 ^b^	3.46 ± 0.02 ^b^	3.01 ± 0.04 ^de^	2.44 ± 0.14 ^d^	0.22 ± 0.02 ^a^	0.85 ± 0.10 ^b^
Mencía	18.98 ± 0.18 ^cd^	10.45 ± 4.14 ^b^	23.61 ± 0.21 ^c^	1.11 ± 0.02 ^bc^	10.15 ± 0.05 ^c^	2.80 ± 0.13 ^a^	1.59 ± 0.01 ^c^	0.84 ± 0.08 ^ab^	0.49 ± 0.01 ^b^	0.92 ± 0.00 ^b^
Mencía	20.55 ± 0.06 ^d^	8.52 ± 0.64 ^b^	33.47 ± 2.62 ^d^	1.00 ± 0.25 ^bc^	18.81 ± 1.18 ^f^	2.68 ± 0.19 ^a^	3.17 ± 0.18 ^e^	0.84 ± 0.02 ^ab^	0.70 ± 0.05 ^b^	0.85 ± 0.17 ^b^
Mencía	22.01 ± 0.10 ^d^	6.22 ± 0.01 ^a^	39.24 ± 0.77 ^ef^	0.23 ± 0.09 ^a^	14.93 ± 0.29 ^de^	1.96 ± 0.03 ^a^	0.93 ± 0.02 ^b^	0.99.1 ± 0.00 ^b^	0.71 ± 0.02 ^bc^	0.62 ± 0.01 ^b^
Jaen	17.52 ± 0.37 ^c^	13.91 ± 0.54 ^bc^	29.64 ± 0.24 ^d^	0.64 ± 0.11 ^a^	9.60 ± 0.24 ^c^	3.76 ± 0.05 ^b^	1.01 ± 0.15 ^b^	4.28 ± 0.17 ^f^	0.64 ± 0.02 ^b^	0.67 ± 0.08 ^b^
Jaen	19.52 ± 0.46 ^cd^	14.11 ± 0.28 ^bc^	42.18 ± 1.21 ^f^	0.46 ± 0.09 ^a^	16.61 ± 0.29 ^e^	3.44 ± 0.08 ^b^	0.59 ± 0.09 ^ab^	2.51 ± 0.04 ^d^	0.16 ± 0.02 ^a^	0.17 ± 0.05 ^a^
Jaen	20.87 ± 1.08 ^d^	17.03 ± 0.80 ^c^	28.18 ± 0.69 ^c^	0.77 ± 0.02 ^b^	15.81 ± 0.21 ^e^	3.56 ± 0.09 ^b^	0.36 ± 0.00 ^a^	2.67 ± 0.03 ^d^	0.24 ± 0.08 ^a^	0.26 ± 0.01 ^a^
Jaen	16.32 ± 0.50 ^c^	7.36 ± 0.22 ^a^	17.14 ± 0.33 ^b^	0.55 ± 0.26 ^a^	15.81 ± 0.21 ^e^	3.69 ± 0.12 ^b^	0.43 ± 0.33 ^a^	3.74 ± 0.16 ^c^	0.14 ± 0.01 ^a^	0.35 ± 0.01 ^a^
Jaen	11.18 ± 0.51 ^b^	10.26 ± 1.37 ^b^	26.06 ± 0.36 ^c^	1.93 ± 0.16 ^d^	10.15 ± 0.12 ^c^	2.18 ± 0.17 ^a^	0.96 ± 0.08 ^b^	1.92 ± 0.07 ^c^	0.56 ± 0.03 ^b^	0.51 ± 0.03 ^a^

Values are presented as mean ± standard deviation (*n* = 2); Means within a column followed by the same superscript letter are not significantly different (Tukey *p* < 0.05).

**Table 2:** ‘Mencía’ and ‘Jaen’ red wines monomeric anthocyanins profile expressed in mg/L.

Wine Samples	D3G	C3G	Pet3G	Peo3G	M3G	D3AG	C3AG	Pet3AG	Peo3AG	M3AG	D3CG	Peo3CG	M3CG
Mencía	1.98 ± 0.01 ^c^	6.31 ± 0.19 ^c^	9.84 ± 0.19 ^c^	10.02 ± 0.10 ^b^	43.44 ± 0.69 ^b^	12.82 ± 0.48 ^f^	1.96 ± 0.15 ^f^	1.00 ± 0.19 ^b^	3.57 ± 0.04 ^e^	5.08 ± 0.21 ^ab^	nd	nd	3.01 ± 0.08 ^b^
Mencía	2.76 ± 0.02 ^d^	5.52 ± 0.07 ^b^	8.19 ± 0.03 ^bc^	13.19 ± 0.06 ^bc^	44.31 ± 0.10 ^b^	3.34 ± 0.01 ^a^	0.52 ± 0.00 ^b^	1.05 ± 0.04 ^b^	2.23 ± 0.01 ^c^	5.84 ± 0.05 ^b^	nd	nd	3.45 ± 0.03 ^bc^
Mencía	4.52 ± 0.14 ^e^	11.04 ± 1.30 ^e^	16.10 ± 0.26 ^d^	16.80 ± 0.18 ^c^	83.43 ± 1.30 ^c^	3.06 ± 0.03 ^a^	nd	1.95 ± 0.01 ^c^	1.99 ± 0.01 ^c^	11.96 ± 0.16 ^c^	nd	nd	5.75 ± 0.06 ^d^
Mencía	1.69 ± 0.02 ^b^	4.58 ± 0.05 ^b^	8.29 ± 0.04 ^bc^	9.28 ± 0.04 ^b^	48.57 ± 0.38 ^b^	2.96 ± 0.02 ^a^	0.49 ± 0.02 ^b^	1.09 ± 0.00 ^b^	1.58 ± 0.05 ^b^	7.31 ± 0.03 ^b^	nd	nd	4.17 ± 0.03 ^c^
Mencía	0.81 ± 0.02 ^a^	1.72 ± 0.01 ^a^	4.12 ± 0.10 ^a^	4.07 ± 0.10 ^a^	36.05 ± 0.54 ^ab^	3.67 ± 0.83 ^b^	0.84 ± 0.01 ^c^	0.89 ± 0.01 ^b^	nd	3.83 ± 0.03 ^a^	nd	nd	1.72 ± 0.02 ^a^
Mencía	2.99 ± 0.29 ^d^	8.00 ± 0.62 ^c^	14.89 ± 1.15 ^d^	14.30 ± 0.14 ^c^	79.00 ± 5.97 ^c^	7.37 ± 0.49 ^d^	1.27 ± 0.06 ^d^	0.56 ± 0.02 ^a^	2.80 ± 0.16 ^d^	10.15 ± 0.75 ^c^	nd	nd	7.52 ± 0.57 ^e^
Mencía	2.09 ± 0.15 ^c^	4.00 ± 0.36 ^b^	6.30 ± 0.56 ^ab^	7.06 ± 0.66 ^a^	28.64 ± 2.40 ^a^	5.01 ± 0.24 ^bc^	0.91 ± 0.10 ^c^	0.79 ± 0.0 ^b^	0.84 ± 0.09 ^a^	2.88 ± 0.30 ^a^	nd	nd	1.89 ± 0.18 ^a^
Mencía	2.04 ± 0.09 ^c^	3.51 ± 0.14 ^ab^	4.98 ± 0.17 ^a^	4.36 ± 0.08 ^a^	30.27 ± 0.37 ^a^	1.97 ± 0.64 ^a^	0.28 ± 0.05 ^a^	0.16 ± 0.08 ^a^	0.76 ± 0.01 ^a^	3.18 ± 0.01 ^a^	nd	nd	2.30 ± 0.07 ^a^
Mencía	1.36 ± 0.00 ^b^	4.63 ± 0.01 ^b^	7.24 ± 0.04 ^bc^	9.86 ± 0.03 ^b^	36.78 ± 0.17 ^ab^	9.75 ± 0.00 ^e^	1.46 ± 0.00 ^de^	0.70 ± 0.01 ^b^	1.48 ± 0.02 ^b^	3.77 ± 0.00 ^a^	nd	nd	2.34 ± 0.02 ^a^
Mencía	1.78 ± 0.09 ^b^	4.71 ± 0.22 ^b^	7.09 ± 0.18 ^bc^	9.24 ± 0.40 ^b^	31.43 ± 1.35 ^a^	8.30 ± 0.46 ^de^	1.14 ± 0.01 ^c^	0.61 ± 0.03 ^a^	1.18 ± 0.09 ^ab^	3.51 ± 0.19 ^a^	nd	nd	2.87 ± 0.14 ^b^
Mencía	2.22 ± 0.01 ^c^	4.31 ± 0.07 ^b^	7.29 ± 0.08 ^bc^	13.14 ± 0.07 ^bc^	35.46 ± 0.42 ^ab^	2.58 ± 0.05 ^a^	0.51 ± 0.02 ^b^	0.89 ± 0.02 ^b^	1.06 ± 0.01 ^a^	2.83 ± 0.03 ^a^	nd	nd	2.94 ± 0.0 3 ^b^
Mencía	1.77 ± 0.26 ^bc^	5.51 ± 0.63 ^b^	7.84 ± 1.94 ^bc^	9.56 ± 3.12 ^b^	34.73 ± 5.59 ^ab^	4.08 ± 0.66 ^b^	0.75 ± 0.15 ^b^	0.70 ± 0.19 ^b^	1.10 ± 0.17 ^a^	3.34 ± 0.45 ^a^	nd	nd	2.63 ± 0.42 ^ab^
Mencía	0.65 ± 0.01 ^a^	8.79 ± 0.03 ^d^	14.70 ± 0.26 ^d^	27.01 ± 0.10 ^e^	133.27 ± 0.59 ^de^	1.83 ± 0.04 ^c^	0.45 ± 0.01 ^b^	0.95 ± 0.02 ^b^	2.64 ± 0.03 ^d^	6.64 ± 0.23 ^b^	nd	nd	2.07 ± 0.06 ^a^
Jaen	1.55 ± 0.01 ^b^	19.70 ± 0.19 ^f^	40.10 ± 0.41 ^g^	29.89 ± 0.06 ^f^	202.51 ± 0.24 ^g^	7.34 ± 0.01 ^d^	0.69 ± 0.01 ^bc^	0.82 ± 0.01 ^b^	8.10 ± 0.03 ^g^	35.56 ± 0.12 ^f^	0.45 ± 0.01 ^a^	3.59 ± 0.17 ^c^	24.72 ± 0.15 ^h^
Jaen	2.02 ± 0.08 ^c^	27.54 ± 0.05 ^g^	41.78 ± 0.03 ^g^	32.22 ± 0.11 ^f^	198.12 ± 0.00 ^g^	13.33 ± 0.13 ^f^	1.68 ± 0.20 ^ef^	0.99 ± 0.03 ^b^	3.07 ± 0.15 ^d^	26.88 ± 0.05 ^e^	0.54 ± 0.15 ^a^	3.91 ± 0.16 ^d^	28.02 ± 0.01 ^i^
Jaen	2.36 ± 0.03 ^c^	10.84 ± 0.02 ^e^	21.52 ± 0.20 ^e^	21.48 ± 0.28 ^d^	123.52 ± 1.17 ^d^	8.34 ± 0.10 ^de^	1.62 ± 0.03 ^e^	0.96 ± 0.01 ^b^	3.65 ± 0.05 ^e^	16.85 ± 0.13 ^d^	nd	1.53 ± 0.14 ^a^	13.68 ± 0.01 ^f^
Jaen	2.07 ± 0.04 ^c^	10.27 ± 1.08 ^de^	27.41 ± 1.63 ^f^	19.98 ± 1.97 ^d^	162.69 ± 4.04 ^f^	6.16 ± 0.10 ^c^	0.74 ± 0.03 ^b^	0.76 ± 0.39 ^b^	7.11 ± 0.21 ^f^	27.40 ± 2.26 ^e^	n.d.	2.12 ± 0.12 ^b^	18.21 ± 0.05 ^g^
Jaen	3.11 ± 0.13 ^a^	8.13 ± 0.23 ^c^	13.66 ± 0.98 ^d^	24.57 ± 2.62 ^e^	141.99 ± 12.8 ^e^	5.96 ± 0.50 ^c^	1.00 ± 0.07 ^c^	0.79 ± 0.12 ^b^	2.67 ± 0.28 ^d^	19.39 ± 0.95 ^d^	n.d.	3.74 ± 0.03 ^cd^	13.64 ± 0.76 ^f^

Values are presented as mean ± standard deviation (*n* = 2); Means within a column followed by the same superscript letter are not significantly different (Tukey *p* < 0.05). Delphinidin-3-glucoside (D3G), cyanidin-3-glucoside (C3G), peonidin-3-glucoside (Peo3G), petunidin-3-glucoside (Pet3G), malvidin-3-glucoside (M3G), delphinidin-3-acetylglucoside (D3AG), cyanidin-3-acetylglucoside (C3AG), peonidin-3-acetylglucoside (Peo3AG), petunidin-3-acetylglucoside (Pet3AG), malvidin-3-acetylglucoside (M3AG), delphinidin-3-coumaroylglucoside (D3CG), peonidin-3-coumaroylglucoside (Peo3CG), malvidin-3-coumaroylglucoside (M3CG).

**Table 3:** Bioclimatic index of Bierzo D.O. and Dão D.O. for 2015 and classes of the viticultural climate of the grape-growing regions.

Bioclimatic Index	Bierzo DO		Dão DO	
Wrinkler index	1549	Moderately cold	1638	Moderately cold
Huglin Heliothermic index	2473	Warm	2217	Temperate warm
Drying Index	−16	Moderately dry	130	Sub-humid
Cool nigh index	7.6	Very cool nights	12.3	Cool nights
Branas Hydrothermic Index	1818	Low risk	2279	Low risk
N. of hot days (>30 °C) ^a^	14		7	
N. of cold nights (<10 °C) ^a^	32		12	

^a^ in August and September months.

**Table 4:** ‘Mencía’ and ‘Jaen’ red wines chromatic characteristics.

Wine Samples	Color Intensity	Hue	Chromatic Characteristics				
	(a.u.)		L*	a*	b*	C*	°h
Mencía	15.36 ± 0.36 ^h^	0.712 ± 0.041 ^b^	68.9 ± 0.0 ^b^	30.63 ± 0.71 ^f^	7.51 ± 0.08 ^fg^	31.54 ± 0.71 ^g^	13.79 ± 0.17 ^f^
Mencía	13.91 ± 0.05 ^g^	0.700 ± 0.002 ^ab^	73.6 ± 0.1 ^e^	27.37 ± 0.54 ^e^	7.82 ± 0.24 ^g^	28.46 ± 0.58 ^f^	15.96 ± 0.17 ^h^
Mencía	13.22 ± 0.01 ^f^	0.750 ± 0.004 ^bc^	73.8 ± 0.1 ^e^	18.43 ± 0.19 ^a^	6.43 ± 0.16 ^e^	19.52 ± 0.24 ^ab^	19.25 ± 0.26 ^i^
Mencía	8.18 ± 0.06 ^b^	0.791 ± 0.003 ^c^	81.3 ± 0.5 ^i^	20.45 ± 0.04 ^b^	7.12 ± 0.00 ^f^	21.66 ± 0.04 ^c^	19.20 ± 0.04 ^i^
Mencía	6.76 ± 0.06 ^a^	0.795 ± 0.011 ^c^	84.3 ± 0.5 ^j^	19.37 ± 0.07 ^ab^	4.95 ± 0.06 ^d^	19.99 ± 0.08 ^b^	14.33 ± 0.11 ^fg^
Mencía	16.23 ± 0.56 ^i^	0.685 ± 0.012 ª	66.7 ± 0.3 ^a^	36.74 ± 0.32 ^i^	6.19 ± 0.10 ^e^	37.26 ± 0.33 ^i^	9.57 ± 0.07 ^d^
Mencía	13.06 ± 0.04 ^f^	0.782 ± 0.004 ^c^	72.2 ± 0.2 ^d^	31.70 ± 0.23 ^fg^	6.22 ± 0.15 ^e^	32.30 ± 0.26 ^g^	11.10 ± 0.19 ^e^
Mencía	10.43 ± 0.04 ^cd^	0.720 ± 0.005 ^b^	83.5 ± 0.7 ^j^	24.12 ± 0.01 ^c^	10.70 ± 0.12 ^h^	26.39 ± 0.04 ^d^	23.94 ± 0.25 ^k^
Mencía	11.95 ± 0.01 ^e^	0.784 ± 0.003 ^c^	76.8 ± 0.3 ^f^	18.37 ± 0.05 ^a^	3.47 ± 0.15 ^b^	18.69 ± 0.08 ^a^	10.71 ± 0.43 ^e^
Mencía	10.57 ± 0.03 ^d^	0.758 ± 0.006 ^bc^	77.5 ± 0.5 ^fg^	26.77 ± 0.08 ^e^	7.94 ± 0.07 ^g^	27.92 ± 0.05 ^ef^	16.53 ± 0.18 ^h^
Mencía	11.05 ± 0.09 ^d^	0.775 ± 0.001 ^c^	78.6 ± 0.3 ^gh^	18.32 ± 0.12 ^a^	4.79 ± 0.08 ^d^	18.93 ± 0.09 ^ab^	14.67 ± 0.33 ^g^
Mencía	9.67 ± 0.02 ^c^	0.731 ± 0.009 ^b^	79.3 ± 0.3 ^h^	25.66 ± 0.07 ^d^	4.46 ± 0.08 ^cd^	26.05 ± 0.06 ^d^	9.86 ± 0.20 ^d^
Mencía	6.12 ± 0.02 ª	0.688 ± 0.017 ^a^	89.1 ± 0.1 ^k^	18.46 ± 0.07 ^a^	7.29 ± 0.01 ^f^	18.95 ± 0.06 ^b^	21.57 ± 0.08 ^j^
Jaen	14.06 ± 0.05 ^g^	0.710 ± 0.002 ^b^	70.6 ± 0.2 ^c^	32.60 ± 0.37 ^g^	1.82 ± 0.02 ^a^	32.65 ± 0.37 ^g^	3.20 ± 0.01 ^a^
Jaen	13.88 ± 0.45 ^g^	0.643 ± 0.032 ^a^	72.1 ± 0.1 ^d^	35.30 ± 0.12 ^h^	3.10 ± 0.11 ^b^	35.45 ± 0.12 ^h^	5.01 ± 0.17 ^b^
Jaen	14.89 ± 0.16 ^h^	0.799 ± 0.037 ^c^	71.2 ± 0.4 ^cd^	25.92 ± 0.09 ^d^	18.14 ± 0.16 ^j^	31.57 ± 0.07 ^g^	35.00 ± 0.15 ^l^
Jaen	15.26 ± 0.07 ^h^	0.714 ± 0.043 ^b^	68.4 ± 0.3 ^b^	34.53 ± 0.40 ^h^	4.27 ± 0.20 ^c^	34.81 ± 0.39 ^h^	7.05 ± 0.25 ^c^
Jaen	12.53 ± 0.14 ^ef^	0.684 ± 0.011 ^a^	79.8 ± 0.0 ^h^	24.82 ± 0.09 ^c^	11.19 ± 0.04 ^i^	27.22 ± 0.06 ^e^	24.29 ± 0.16 ^k^

The values are presented as mean ± standard deviation; Means within a column followed by the same superscript letter are not significantly different (Tukey *p* < 0.05). L*—lightness, a*—redness, b*—yellowness, C*—chroma; °h—hue angle, a.u.—absorbance units.

**Table 5:** ‘Mencía’ and ‘Jaen’ red wines total anthocyanins, colored anthocyanins, polymeric, and total pigments.

Wine Samples	Total Anthocyanins (mg/L)	Colored Anthocyanins (a.u.)	Total Pigments (a.u.)	Polymeric Pigments (a.u.)
Mencía	277 ± 2 ^e^	2.83 ± 0.05 ^a^	20.00 ± 2.29 ^cd^	4.78 ± 0.11 ^g^
Mencía	257 ± 8 ^d^	4.23 ± 0.08 ^b^	17.88 ± 0.43 ^cd^	2.83 ± 0.07 ^fg^
Mencía	243 ± 4 ^cd^	4.80 ± 0.05 ^c^	19.04 ± 0.93 ^cd^	1.75 ± 0.06 ^c^
Mencía	208 ± 2 ^b^	2.28 ± 0.01 ^a^	18.53 ± 0.21 ^cd^	1.72 ± 0.03 ^c^
Mencía	190 ± 6 ^b^	2.15 ± 0.14 ^a^	9.80 ± 0.71 ^ab^	1.19 ± 0.04 ^b^
Mencía	333 ± 1 ^f^	5.20 ± 0.17 ^cd^	20.65 ± 0.007 ^cd^	3.07 ± 0.02 ^g^
Mencía	293 ± 6 ^e^	3.87 ± 0.02 ^b^	16.77 ± 2.57 ^c^	2.42 ± 0.04 ^e^
Mencía	203 ± 1 ^b^	4.34 ± 0.00 ^bc^	12.42 ± 0.00 ^b^	0.90 ± 0.01 ^a^
Mencía	254 ± 9 ^cd^	2.98 ± 0.14 ^a^	11.62 ± 0.14 ^b^	2.52 ± 0.014 ^e^
Mencía	232 ± 9 ^c^	3.03 ± 0.03 ^a^	10.61 ± 0.86 ^ab^	2.20 ± 0.04 ^d^
Mencía	231 ± 3 ^c^	3.05 ± 0.11 ^a^	10.66 ± 0.07 ^ab^	2.24 ± 0.09 ^d^
Mencía	236 ± 2 ^cd^	2.88 ± 0.01 ^a^	10.40 ± 0.14 ^ab^	2.05 ± 0.001 ^d^
Mencía	147 ± 8 ^a^	2.36 ± 0.00 ^a^	7.37 ± 0.00 ^a^	0.93 ± 0.025 ^a^
Jaen	515 ± 9 ^i^	5.04 ± 0.15 ^cd^	31.41 ± 0.71 ^e^	2.02 ± 0.06 ^d^
Jaen	525 ± 8 ^i^	5.32 ± 0.86 ^cd^	21.56 ± 1.07 ^d^	2.38 ± 0.01 ^e^
Jaen	382 ± 5 ^g^	3.69 ± 0.22 ^b^	27.52 ± 0.21 ^e^	2.74 ± 0.05 ^f^
Jaen	434 ± 1 ^h^	5.89 ± 0.18 ^d^	38.08 ± 1.00 ^f^	1.71 ± 0.10 ^c^
Jaen	381 ± 7 ^g^	2.96 ± 0.01 ^a^	16.97 ± 0.14 ^c^	1.77 ± 0.01 ^c^

The values are presented as mean ± standard deviation; Means within a column followed by the same superscript letter are not significantly different (Tukey *p* < 0.05). a.u.—absorbance units.
